# Reduced Insulin/Insulin-Like Growth Factor Receptor Signaling Mitigates Defective Dendrite Morphogenesis in Mutants of the ER Stress Sensor IRE-1

**DOI:** 10.1371/journal.pgen.1006579

**Published:** 2017-01-23

**Authors:** Yehuda Salzberg, Andrew J. Coleman, Kevin Celestrin, Moran Cohen-Berkman, Thomas Biederer, Sivan Henis-Korenblit, Hannes E. Bülow

**Affiliations:** 1 Department of Genetics, Albert Einstein College of Medicine, Bronx, New York, United States of America; 2 The Mina and Everard Goodman Faculty of Life Sciences, Bar-Ilan University, Ramat-Gan, Israel; 3 Department of Neuroscience, Tufts University School of Medicine, Boston, MA, United States of America; 4 Dominick P. Purpura Department of Neuroscience, Albert Einstein College of Medicine, Bronx, New York, United States of America; The University of Texas Health Science Center at Houston, UNITED STATES

## Abstract

Neurons receive excitatory or sensory inputs through their dendrites, which often branch extensively to form unique neuron-specific structures. How neurons regulate the formation of their particular arbor is only partially understood. In genetic screens using the multidendritic arbor of PVD somatosensory neurons in the nematode *Caenorhabditis elegans*, we identified a mutation in the ER stress sensor IRE-1/Ire1 (inositol requiring enzyme 1) as crucial for proper PVD dendrite arborization *in vivo*. We further found that regulation of dendrite growth in cultured rat hippocampal neurons depends on Ire1 function, showing an evolutionarily conserved role for IRE-1/Ire1 in dendrite patterning. PVD neurons of nematodes lacking *ire-1* display reduced arbor complexity, whereas mutations in genes encoding other ER stress sensors displayed normal PVD dendrites, specifying IRE-1 as a selective ER stress sensor that is essential for PVD dendrite morphogenesis. Although structure function analyses indicated that IRE-1’s nuclease activity is necessary for its role in dendrite morphogenesis, mutations in *xbp-1*, the best-known target of non-canonical splicing by IRE-1/Ire1, do not exhibit PVD phenotypes. We further determined that secretion and distal localization to dendrites of the DMA-1/leucine rich transmembrane receptor (DMA-1/LRR-TM) is defective in *ire-1* but not *xbp-1* mutants, suggesting a block in the secretory pathway. Interestingly, reducing Insulin/IGF1 signaling can bypass the secretory block and restore normal targeting of DMA-1, and consequently normal PVD arborization even in the complete absence of functional IRE-1. This bypass of *ire-1* requires the DAF-16/FOXO transcription factor. In sum, our work identifies a conserved role for *ire-1* in neuronal branching, which is independent of *xbp-1*, and suggests that arborization defects associated with neuronal pathologies may be overcome by reducing Insulin/IGF signaling and improving ER homeostasis and function.

## Author Summary

Sensory neurons sample their environment through highly branched structures termed dendritic arbors or trees. The precise patterning of dendritic arbors is important for the proper functioning of the nervous system, and evidence indicates an involvement of sensory neurons in neuropsychiatric disease such as autism spectrum disorders. The unfolded protein response is a cellular process that ensures and maintains a functional protein-folding environment in the cell’s endoplasmic reticulum, and is compromised in a number of neurodegenerative conditions. Recently, this process has also been implicated in dendrite patterning. We discovered that the function of the unfolded protein response in dendrite patterning is evolutionarily conserved from the roundworm *C*. *elegans* to mammals. Specifically, dendrites in both worms and mammals require the function of a conserved enzyme with both kinase and ribonuclease activity, which acts as a sensor for the unfolded protein response. Importantly, we find that loss of this enzyme can be bypassed by reducing the signaling through the insulin-like growth factor receptor. Our findings reveal a new way of bypassing defects in the unfolded protein response during dendrite development.

## Introduction

During their development neurons can form complex dendritic branching patterns. The specific arbor morphologies of different neuron types are thought to have evolved to mediate the acquisition and processing of distinct inputs [1]. Defective arbor morphologies in brain neurons are a common cellular symptom in many neuropsychiatric and neurodegenerative diseases [2–4].

Dendritic arbor growth requires the accurate orchestration of numerous cellular events that occur concomitantly at a distance from the neuronal cell body and integrate dramatic membrane extension, local protein translation and processing, vesicular transport, shifts in cytoskeleton dynamics and elevated metabolic activity. How neurons control these various processes at the genetic and molecular level remains only partially understood [5–7].

Our understanding of dendrite arbor morphogenesis has advanced significantly through the study of peripheral mechanosensory arbor development in the fly *Drosophila melanogaster* and the nematode *Caenorhabditis elegans*. In *Drosophila*, larval *da* (*dendrite arborization*) neurons are grouped into four classes according to the degree of arbor complexity [6, 8]. Screens for *da* dendrite defects have identified many genes that control arborization, such as transcription factors, membrane receptors and their ligands, integrins, vesicular transport factors and cell adhesion molecules [5, 6, 8]. Recently, the polymodal sensory neuron PVD in *C*. *elegans* with its characteristic multidendritic arbor has become a model neuron for the study of dendrite morphogenesis [9, 10]. Genetic work on the formation of the repetitive PVD menorah-shaped dendritic units has identified several genes not implicated before in dendrite morphogenesis, including roles for fusogens [11], the LRR-type receptor *dma-1* [12], the fam151 family member *mnr-1*/menorin [13, 14], the secreted leukocyte cell-derived chemotaxin 2 *lect-2/chondromodulin II* [15, 16], and the furin-like protease *kpc-1* [17–19].

The endoplasmic reticulum (ER) is the primary cellular site for secretory protein and lipid biosynthesis, both of which are essential for proper cellular function. In agreement, disruption of ER homeostasis is associated with pathologies such as neurodegenerative disorders [20–22]. To prevent deleterious outcomes of perturbed ER homeostasis, a cellular program called the Unfolded Protein Response (UPR) is triggered at times of increased load on the ER (i.e. ER stress) to ensure that ER homeostasis is retained regardless of the dynamic nature of cellular demand [23]. In mammalian cells (as well as in *C*. *elegans*), the UPR is composed of three pathways that are initiated by distinct ER stress sensors: inositol-requiring enzyme 1 (IRE1), protein kinase RNA (PKR)-like ER kinase (PERK) and activating transcription factor-6 (ATF6). IRE1 is the most ancient of the UPR sensors, being conserved from yeast to humans, and bears both kinase and ribonuclease activities [24]. Upon its activation, IRE1 undergoes autophosphorylation and oligomerization into multimers [25, 26]. In its oligomeric state it removes an intron from *xbp-1* (X-box binding protein-1) mRNA through unconventional splicing allowing the translation of an activated form of the XBP-1 transcription factor. This activated transcription factor induces the expression of chaperones, ERAD components and other ER auxiliary factors to rebalance ER capacity [27, 28]. The UPR, and specifically the *ire-1/xbp-1* arm of the UPR, is important even under normal physiological conditions (i.e. in the absence of induced ER stress), as perturbations in this pathway impair secretory protein metabolism [29].

Additional *xbp-1* independent functions of *ire-1* are also known. These include activation of the cell death machinery [30–32], induction of autophagosomes [33], induction of a cellular anti-oxidant response [34] and degradation of ER-localized mRNAs that encode secreted and membrane proteins through the RIDD (regulated Ire1-dependent decay) pathway [35]. Recent *in vitro* work using the yeast Ire1 has suggested that RIDD activity can be mediated by IRE1 even in its monomeric state [36].

Here, we demonstrate that IRE1’s role in dendrite arborization is conserved during evolution from *C*. *elegans* to mammals. We show that in *C*. *elegans ire-1* deficiency elicits a secretory block in the PVD neuron that interferes with the targeting of the DMA-1 receptor to the plasma membrane, strengthening similar results by Wei *et al*. [37]. We further reveal that this trafficking block, which does not occur in *xbp-1* mutants, can be bypassed by reducing insulin/IGF1 signaling to restore normal arbor architecture. Altogether, this work assigns a conserved role for IRE-1 function in neuronal development and demonstrates that activation of alternative ER homeostasis-promoting pathways can counteract and prevent the deleterious consequences of compromised ER homeostasis on neuronal development.

## Results and Discussion

### The UPR sensor *ire-1/IRE1* is required for dendrite morphogenesis in *C*. *elegans* and in mammals

**Fig 1:**
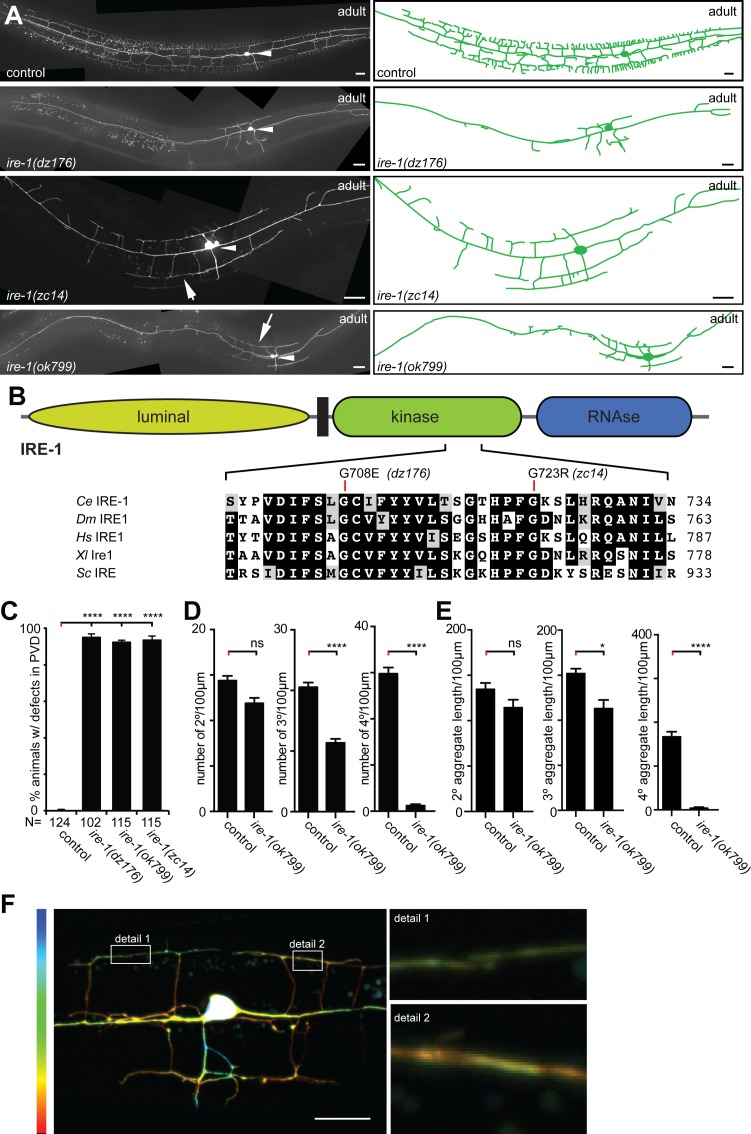
The inositol requiring enzyme 1 (IRE-1) is required in PVD neurons to shape sensory dendrites. A. Fluorescent micrographs with tracings (right panels) of PVD in the genotypes indicated (PVD visualized by the *wdIs52* transgene (*Is[F49H12*.*9*::*GFP]*). Arrowheads indicate cell bodies and arrows areas with defects in self-avoidance. Anterior is to the left in all panels and ventral down; scale bars indicate 20 μm. B. Schematic of the inositol-requiring enzyme 1 (IRE-1) with a multiple sequence alignment of part of the kinase domain (accession numbers: *C*. *elegans (Ce)*: Q09499.2, *D*. *melanogaster (Dm)*: ABW08704.1, *H*. *sapiens (Hs)*: HGNC: 3449, *X*. *laevis (Xl)*: AAH73092.1, *S*. *cerevisiae (Sc)*; NP_011946.1). Location of missense alleles and amino acid position (right) is indicated. C. Quantification of defects in the genotypes indicated. Defects are defined as absence of complete menorah-like dendrites between the vulva and the anterior end of the animals. D–E. Quantification of secondary, tertiary, and quaternary branch numbers (D.) and aggregate length (E.). Data are represented as mean ± SEM. Statistical comparisons were performed using one-way ANOVA with the Tukey correction. Statistical significance is indicated (*p = 0.05, **p = 0.01, ****p = 0.0001). n = 20 wild-type control animals (1295 dendritic branches), and n = 20 *ire-1(ok799)* mutant animals (489 dendritic branches). F. Maximum-intensity projection of an *ire-1(dz176)* mutant animal in which each individual optical section is labeled in a different color (7 sections of 0.6 μm each). Warmer and colder colors indicate more medial and lateral sections of the animal, respectively. Overlapping tertiary dendrites (within boxes and shown in magnification) appear in the same focal plane (based on the same color). The width of dendritic branches is approximately 200 nm [9], suggesting that tertiary dendrites in *ire-1(dz176)* mutants are closely apposed (within 0.6 μm) or directly touching.

The dendrites of the polymodal somatosensory PVD neurons are stereotypically patterned, by the consecutive branching of secondary, tertiary, and quaternary branches from primary dendrites that exit the PVD cell body on either side both in an anterior and in a posterior direction (Fig 1A). In concordance with a recent report [37], we isolated a mutant allele of *ire-1*, which encodes the *C*. *elegans* homolog of the inositol requiring enzyme 1 (IRE1) in a screen for genes required for PVD morphogenesis [13]. The *ire-1(dz176)* allele changes Glycine 708, a residue that is located in an alpha helix of the kinase domain and conserved from yeast to humans (Fig 1A and 1B). The PVD phenotypes were shared with another missense allele (*zc14*), which also changed a perfectly conserved G723 in the kinase domain, as well as the deletion allele *ok799* (Fig 1A–1C). Mutant phenotypes were transgenically rescued by both a wild-type copy of *ire-1* (S1A–S1C Fig) and expression of a cDNA pan-neuronally or in PVD neurons, but not in the intestine or hypodermis (skin) (S1D Fig). These findings complement previous mosaic studies [37], and together strongly argue for a cell-autonomous function of *ire-1*.

As reported previously, *ire-1* mutants formed dendrites with quaternary branches only in the area proximal to the PVD cell body, and gradually became less developed as their distance from the cell body increased both anteriorly and posteriorly (Fig 1A) [37]. We extended these observations in morphometric analyses, which showed a reduction both in the number and aggregate length of secondary, tertiary and quaternary branches in the presumptive *ire-1(ok799)* null mutant (Fig 1D and 1E; S1E Fig). In addition, we discovered a self-avoidance defect in *ire-1* mutants, where adjacent tertiary dendrites failed to retract upon mutual contact, thereby eliminating the characteristic gaps between them (Fig 1F).

**Fig 2:**
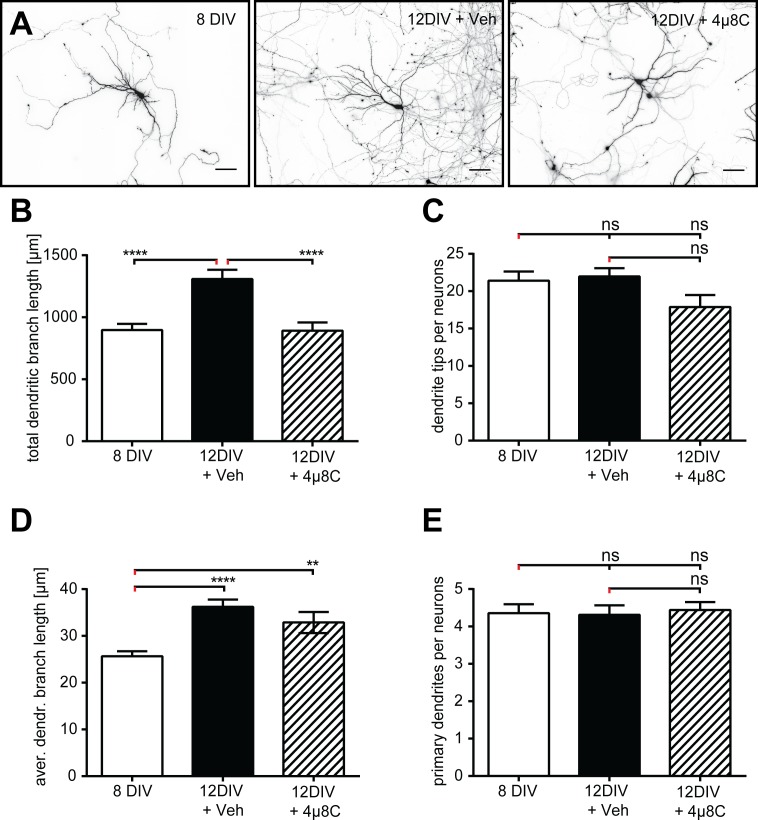
IRE-1 is required for dendrite growth in dissociated hippocampal neurons. A. Images of dissociated hippocampal neurons at indicated days *in vitro* (DIV) and either DMSO-vehicle control treated (Veh) or treated with an inhibitor of IRE-1 (4μ8C). Scale bars indicate 50 μm. B. Total dendritic branch length increases from 8 to 12 DIV in control neurons and this increase is blocked in 4μ8C-treated cultures. C. Dendrite tip number did not increase with development, however 4μ8C-treated cultures exhibited a non-significant trend towards reduced tip number reflecting decreased dendrite complexity. D. Average dendritic branch length increased from 8 to 12 DIV, and this was not affected by 4μ8C treatment. E. Primary dendrite number was stable from 8 to 12 DIV and was not affected by 4μ8C treatment. 8 DIV, 42 neurons; 12 DIV + Veh, 42 neurons; 12 DIV + 4μ8C, 41 neurons. All data are from three independent experiments. **p < .01; ***p < .001; ****p < .0001; ns, not significant (p > 0.05).

Since the role of IRE1 in dendrite patterning in mammals had never been addressed before, we investigated whether IRE1 serves an evolutionarily conserved function during dendrite patterning in mammals. We studied dendrite morphogenesis in dissociated rat hippocampal cultures and measured changes in dendrite length and complexity after 8 and 12 days *in vitro* (DIV), a time period during which dendrites undergo dynamic growth (Fig 2A). During this time window, neurons in culture were either treated with vehicle or the IRE1-specific inhibitor 4μ8C [38]. Vehicle-treated neurons showed the expected developmental increase in total dendritic branch length from 8 to 12DIV (Fig 2B; 8DIV, 897 +/- 50 μm, n = 42 vs. 12DIV+veh, 1308 +/- 75 μm, n = 42; p<0.0001). In contrast, neurons treated from 8DIV onwards with 50 μM of the specific IRE1 RNAse inhibitor 4μ8C did not show this developmental increase in total dendritic branch length (Fig 2B, 8DIV vs. 12DIV+4μ8C, 893 +/- 66 μm, n = 41; p = 0.99). In neurons treated with the IRE1 inhibitor, there was a trend towards fewer dendrite tips as compared with vehicle treated neurons (Fig 2C; 12DIV+veh, 22 +/- 1.1 tips vs. 12DIV+4μ8C, 18 +/- 1.6 tips; p = 0.078), consistent with the correlation of shorter total dendritic branch length with fewer dendritic tips [39]. IRE1 inhibition did not restrict the developmental increase in average dendritic branch length, supporting the notion that this aspect of dendrite differentiation was not impaired (Fig 2D; 8DIV, 26 +/- 1.1 μm vs. 12DIV+veh, 36 +/- 1.6 μm; p<0.0001; 8DIV vs. 12DIV+4μ8C, 33 +/- 2.3 μm, p<0.001). Importantly, the effect of IRE1 inhibition was specific to higher order branches and did not alter the number of primary branches (Fig 2E), similar to the effects observed in PVD dendrites of *ire-1* mutants in *C*. *elegans* (Fig 1D and 1E). We conclude that IRE-1 serves a conserved role in dendritic dendrite morphogenesis under normal physiological conditions, and in the absence of external induction of ER stress. Thus, our studies in rats and *C*. *elegans* provide the first example for a conserved developmental function of the ire1 stress sensor in neural development. This adds to a growing body of literature, based on knockout approaches in mice, that show functions for the unfolded protein response during liver development [40–42], as well as in the development of antibody-producing B cells [43] and secretory cells of the pancreas [44].

### The nuclease activity of IRE-1 is required for PVD dendritic branching

**Fig 3:**
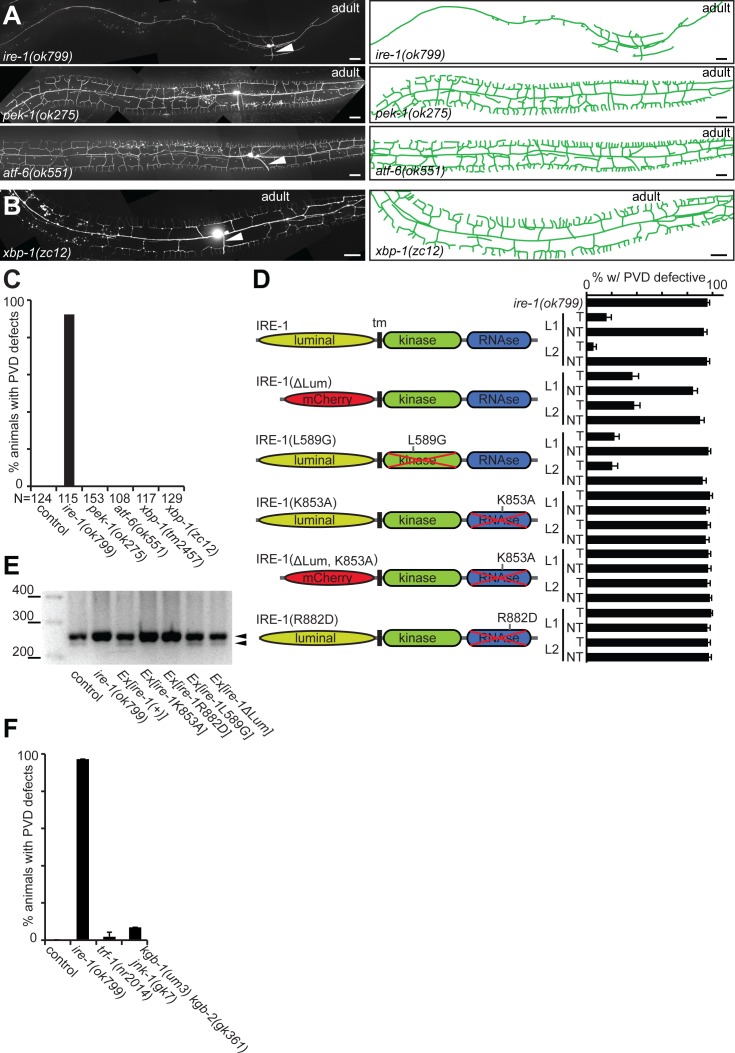
IRE-1 requires its nuclease activity independently of canonical UPR pathways. A–B. Fluorescent micrographs of mutant animals with tracings (right panels) of the genotypes indicated. Anterior is to the left and ventral down in all images. Arrowheads indicate the PVD axons and scale bars 20 μm. C. Quantification of animals with PVD defects in animals of the indicated genotypes. D. Structure function analyses of *ire-1(ok799)* mutant animals with the constructs as indicated. All constructs were transgenically expressed under control of the heterologous pan-neuronal *Prgef-1* promoter. Transgenic (T) and non-transgenic siblings (NT) were analyzed in parallel in different transgenic lines (L1, L2, etc.). tm: transmembrane domain. E. *xbp-1* splicing assay using RNA from the genotypes indicated (see Materials and Methods for details). Successful splicing is evidenced by the presence of a band that is slightly smaller (237 nt) than the unspliced band (260 nt), both indicated by black arrowheads. The size of selected bands in a molecular marker is shown. F. Quantification of animals with PVD defects in animals of the indicated genotypes.

The IRE-1 protein is composed of a luminal unfolded protein sensor domain and a cytosolic bifunctional active site, comprising a kinase and a ribonuclease domain. Our mutants in the kinase domain, as well as mutants identified by Wei *et al*. [37] suggested that both domains may be important for *ire-1* function. To further investigate this notion, we generated mutant versions of IRE-1, defective in each of these domains, and conducted rescue experiments in *ire-1* mutants. We found that expression of a mutant where the ER luminal domain, thought to serve as an unfolded protein receptor [45], had been replaced by red fluorescent protein (mCherry), rescued PVD morphology in *ire-1* mutant animals, although not as efficiently as the full length transgene (Fig 3D). In contrast, expression of the ribonuclease-deficient mutant version IRE-1^K853A^, affecting a highly conserved residue in the putative nuclease active site [46] and completely devoid of any detectable *xbp-1* splicing activity (Fig 3E), failed to rescue PVD arbor morphology in *ire-1* mutant animals (Fig 3D). This implies that IRE-1 nuclease activity is necessary for dendrite morphogenesis. In addition, we expressed IRE-1^L589G^, an IRE-1 transgene harboring a mutation analogous to the yeast ire1p mutation L745G, which alters the specificity of the ATP binding site in the kinase domain of the protein [47]. In contrast to the yeast studies, the ribonuclease activity of IRE-1^L589G^ appeared intact in an *xbp-1* splicing assay (Fig 3E). We found that expression of IRE-1^L589G^ also rescued the arborization defects in PVD sensory dendrites (Fig 3D). Collectively, our rescue studies show that PVD development requires the ribonuclease activity of IRE-1. This conclusion is consistent with the defective PVD arborization phenotype previously observed in *ire-1(wy762)* mutants, in which a conserved residue in the endoribonuclease domain of the protein has been altered [37]. In addition, kinase activity is likely required, because three mutant alleles of *ire-1* (*dz176*, *zc14*, this study; *wy782*, [37]) that result in substitutions of distinct conserved residues in the kinase domain of the protein, display a defective PVD arborization phenotype.

### PVD dendritic branching is independent of *xbp-1*

In addition to IRE-1, metazoans have at least two distinct additional sensors of ER stress, the *pek-1*/PERK kinase and the *atf-6*/ATF6 transcription factor [48]. Interestingly, PVD development proceeds normally in *pek-1*/PERK and *atf-6*/ATF6 mutants, demonstrating that they do not individually serve a critical role in PVD dendrite morphogenesis, and pointing at a unique function of IRE-1 (Fig 3A and 3C)[37].

To gain insight in the downstream effectors of IRE-1 signaling, we focused on the processing of *xbp-1* mRNA through unconventional splicing by IRE-1, the best known activity of IRE-1 [27, 28]. Interestingly, two different *xbp-1* mutant alleles, *zc12* and *tm2457* displayed a PVD arbor that was indistinguishable from wild type animals (Fig 3B and 3C), suggesting that IRE-1 can function through *xbp-1*-independent activities in patterning PVD dendrites.

Known *xbp-1*-independent functions of *ire-1* include activation of the TRAF2 and JNK kinase signaling cascade [30, 49], and degradation of ER-localized mRNAs that encode secreted and membrane proteins through the RIDD (regulated Ire1-dependent decay) pathway [35]. We found that PVD arborization remained normal upon depletion of the *C*. *elegans* TRAF2 homolog *trf-1* or concomitant depletion of all three *C*. *elegans jnk-1*-related kinases (Fig 3F) suggesting that neither pathway plays non-redundant roles in PVD morphogenesis. To directly explore whether RIDD is the mechanism by which IRE-1 controls PVD arborization, we sought another way to maintain RIDD activity in *ire-1* mutants while compromising *xbp-1* splicing activity. A mutation in the yeast yIre1 protein, R1087D, uncouples the two nuclease activities of ire1p in yeast by impairing *xbp-1* splicing while leaving RIDD activity intact [36]. The analogous mutation in worms, IRE-1^R882D^, failed to rescue PVD architecture (Fig 3D). Since *xbp-1* function is not required for PVD morphogenesis, we suggest that *C*. *elegans* IRE-1^R882D^ mutant protein may not discriminate between *xbp-1-*related and unrelated nuclease activities. Thus, among the known *xbp-1* independent activities of IRE-1, RIDD remains the most likely to mediate PVD dendrite arborization. This conclusion supports experiments where mosaic knock out of an essential *xrn-1* RNA endonuclease, believed to be part of the RIDD pathway, produced low penetrance defects in PVD neurons [37].

### The neuronal receptor DMA-1 mislocalizes to the PVD cell body in *ire-1* mutants

**Fig 4:**
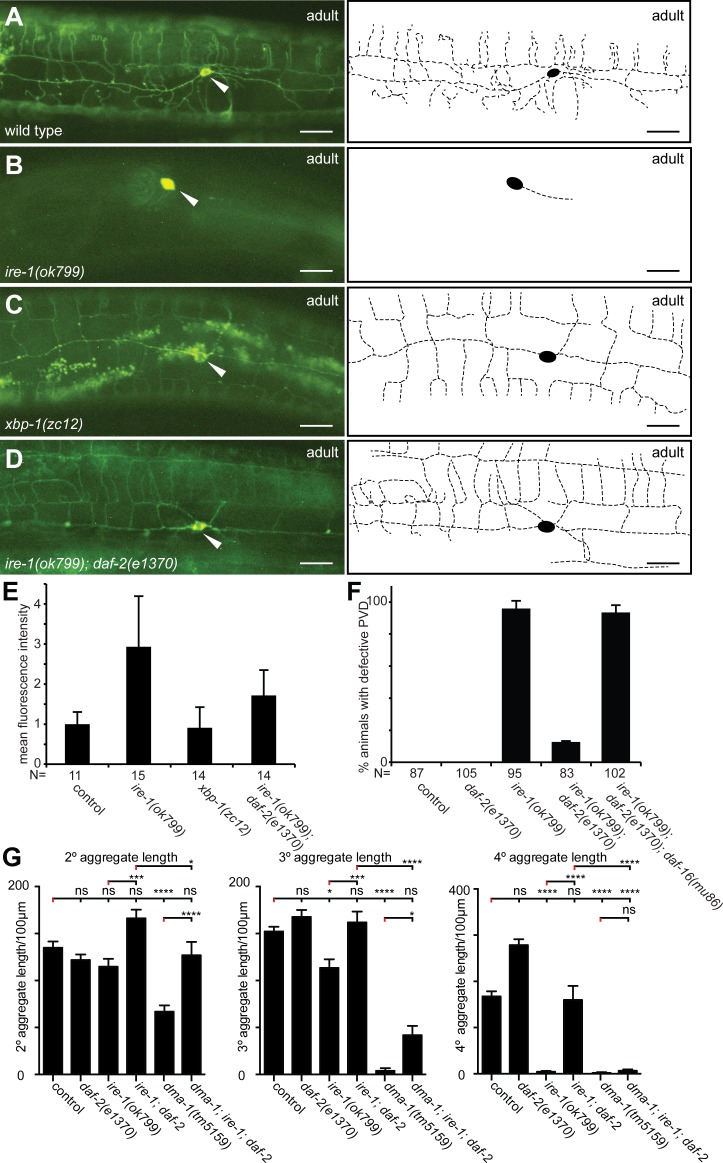
Reducing DAF-2/IIS signaling mitigates the secretory block in *ire-1* mutant animals. A–D. Fluorescent micrographs of animals transgenically expressing a translational DMA-1::GFP fusion [12] in the genotypes indicated. Anterior is to the left in all panels and ventral down. Arrowheads indicate the PVD cell body and scale bars 20 μm. E. Quantification of mean fluorescent intensity of the PVD cell body in different genetic backgrounds. F. Quantification of defects in PVD in animals in the indicated genotypes. G. Quantification of branch number in *daf-2(e1370)*, *ire-1(ok799)*, *dma-1(tm5159)* single-, double- and triple-mutant animals. Data are represented as mean ± SEM. Statistical comparisons were performed using one-way ANOVA with the Tukey correction, and statistical significance is indicated (*p = 0.05, **p = 0.01, ***p = 0.001, ****p = 0.0001; ns, not significant [p > 0.05]). n = 20 wild-type controls (1295 dendritic branches), n = 20 *daf-2(e1370)*mutant animals (1046 dendritic branches), n = 20 *ire-1(ok799)* mutant animals (489 dendritic branches), n = 20 *dma-1(tm5159)* mutant animals (381 dendritic branches), n = 20 *ire-1(ok799); daf-2(e1370)* mutant animals (1100 dendritic branches), n = 19 *dma-1(tm5159); ire-1(ok799); daf-2(e1370)* mutant animals (470 dendritic branches). Data for wild-type control and *ire-1(ok799)* animals are identical to those in Fig 1 and are shown for comparison only.

Recently, it was shown that even under normal growth conditions (i.e. without artificially-induced ER stress) *ire-1* mutants display defects in the metabolism of secretory proteins [29]. One central protein located on the cell membrane of PVD and essential for proper dendrite branching is the DMA-1 leucine rich repeat transmembrane receptor [12]. In concordance with a recent report [37] we found that a DMA-1::GFP reporter primarily localized to the cell body of *ire-1* mutant animals (Fig 4B and 4E). In contrast, in wild-type animals, the DMA-1::GFP reporter localized both to the cell body as well as to the entire PVD arbor, throughout the primary, secondary, tertiary and quaternary branches (Fig 4A and 4E). Importantly, although the primary branch of the PVD dendrite is always present and extends along the body of *ire-1*-deficient animals (Fig 1A), DMA-1::GFP expression was restricted to the cell body and was not detected on the plasma membrane of the primary branch of PVD (Fig 4B). This suggests that DMA-1 is specifically required for patterning of the secondary, tertiary and quaternary branches in *ire-1-*deficient animals. This further suggests that the DMA-1::GFP localization defect in *ire-1* mutants precedes the PVD patterning defect. Collectively, these observations suggest that DMA-1 fails to shuttle properly through the secretory pathway, resulting in patterning defects of higher order branches of the PVD dendrite.

Intriguingly, *xbp-1* mutants displayed completely normal DMA-1::GFP staining of the entire PVD dendrite similar to wild-type animals (Fig 4C), and did not accumulate DMA-1::GFP in the soma like *ire-1* mutants (Fig 4E). The proper localization of DMA-1::GFP in *xbp-1* mutants contrasts with its mislocalization in *ire-1* mutants (Fig 4A–4C and 4E), but is consistent with the PVD arborization architecture seen in the respective mutants (Fig 3A–3C). This finding was surprising, given that loss of *xbp-1* has been shown to perturb ER homeostasis and interfere with secretory protein metabolism [29]. Based on the trafficking defects in *ire-1* mutant animals, and the finding that the basal activity of the UPR in PVD itself is largely dependent on DMA-1 expression, it has been suggested that the failure of DMA-1::GFP to reach the plasma membrane is a consequence of a folding challenge of DMA-1 itself [37]. However, we point out that although in many cases functional *xbp-1* is also required for the trafficking and maturation of other secreted and transmembrane proteins [29, 50], it was not required for DMA-1 trafficking to the plasma membrane, and PVD morphogenesis is normal in *xbp-1* mutants. Thus, DMA-1 can traffic to the plasma membrane and support PVD dendrite morphogenesis even under the unfavorable proteostatic conditions in the ER of *xbp-1*-deficient animals. This suggests that DMA-1 may not have an intrinsic tendency to fold improperly and that the DMA-1 trafficking defect is more likely a reflection of a general overload and perturbed function of the ER in the PVD neuron that lacks *ire-1*. A possible explanation for the differences between *ire-1* mutants and *xbp-1* mutants is that ER homeostasis and function is less compromised in *xbp-1-*deficient animals compared to *ire-1*-deficient animals [29]. Thus, the DMA-1-dependent activation of the UPR in PVD suggested by Wei *et al*. [37] may be an indirect consequence of DMA-1 promoting dendrite morphogenesis and expansion, both of which require the synthesis of membranes proteins and lipids and impose a significant biosynthetic load on the ER. This ‘capacity model’ is also consistent with the observation that overexpression of spliced *xbp-1* or its target, the ER-localized heat shock protein HSP-4/BiP/grp78 can bypass the requirement for *ire-1* and rescue the morphological defects and the DMA-1::GFP secretion defects in *ire-1* mutants [37]. Altogether, our report adds to a growing number of recent works delineating an *xbp-1*-independent branch of the *ire-1* pathway [29, 35, 40, 51, 52].

### Reduced insulin/IGF-1 signaling suppresses the PVD defects of *ire-1* mutants

If the failure to form menorahs in *ire-1* mutants is a result of a block in the secretory pathway in the PVD neuron then conditions that release the secretory block in *ire-1* mutants should restore PVD arborization. One way to overcome the secretory block in *ire-1* mutants is by activating the FOXO transcription factor DAF-16, which is inhibited by the insulin/IGF-1 signaling (IIS) pathway [52]. Indeed, reducing IIS in animals through a mutation in their *daf-2* gene, the only insulin-like growth factor receptor in *C*. *elegans*, resulted in reduced accumulation of DMA-1::GFP in the cell body and redistribution to the plasma membrane of PVD in *ire-1* mutants (Fig 4D and 4E). Consistent with the restored localization of DMA-1::GFP expression pattern in the PVD neuron, we found that PVD dendrite morphogenesis defects in *ire-1; daf-2* double mutants were completely reversed and PVD arbors of double mutants were indistinguishable from wild type animals (Fig 4F). This finding was further corroborated by morphometric analyses. We found that the reduced length of secondary, tertiary and quaternary branches in *ire-1* mutants was suppressed upon reduced DAF-2/IIS signaling (Fig 4G). Moreover, this suppression was largely (although not completely) *dma-1-*dependent, because *dma-1* appeared epistatic in a *dma-1; ire-1; daf-2* triple mutant (Fig 4G).

The physiological consequence of reduced DAF-2/IIS signaling, including improving ER homeostasis in *ire-1*-deficient animals [52], in many cases depends on the activation of the transcription factor DAF-16/FOXO. We found that *daf-16; ire-1; daf-2* triple mutant animals showed the same frequency of PVD defects as *ire-1* single mutants, indicating that the suppression of defects in *ire-1* mutants by loss of *daf-2* insulin signaling was entirely dependent on *daf-16* activation (Fig 4F). In other words, the defects in dendrite morphogenesis of *ire-1* mutants can be rescued by compromising DAF-2/IIS signaling in a *daf-16/FOXO-*dependent manner.

Our finding that trafficking of a DMA-1::GFP reporter is restored in *daf-2/IIS* mutants suggests that (1) different approaches can be used to relieve the secretory block in *ire-1* mutants, and (2) are consistent with previous observations that attenuation of IIS can result in favorable effects on proteostasis, ER homeostasis, organismal health and survival in *C*. *elegans*, as well as other organisms [53, 54]. Similarly, activation of the IIS regulated transcription factor DAF-16/FOXO3A in *ire-1*-deficient cells can bypass the requirement of the canonical *ire-1/xbp-1* pathway for the maintenance of ER homeostasis, and improve both ER homeostasis and restoration of normal secretory protein trafficking in worms and mammalian cells [52]. Thus, our findings may provide a mechanistic explanation for observations in several studies showing that neurons grow and function better under reduced IIS conditions [55–57], and expands this notion to include dendritic arbor morphogenesis. Since the improvement on DMA-1::GFP trafficking and dendrite morphology were dependent on activation of the DAF-16/FOXO transcription factor, the activation of this pathway by alternative cues including starvation as well as a variety of cytotoxic stresses (e.g. heat-shock and oxidative stresses), which directly or indirectly activate DAF-16, hold the potential to recover PVD dendrite morphogenesis in the absence of a properly functioning UPR.

In summary, our results establish that the function of the IRE-1 UPR sensor in neuronal patterning is conserved from invertebrates to mammals. Our findings demonstrate that promoting ER homeostasis, e.g. by reducing IIS, can overcome morphological defects in neuronal patterning. This underscores the importance of discovering and investigating new approaches that can bypass excessive ER stress. Given the conservation of the role of the UPR in dendrite branching and morphogenesis from *C*. *elegans* to mammals, as well as the conservation of the proteostasis-promoting effects of the IIS pathway, these findings may offer novel approaches for treatment of neurodegenerative disorders.

## Materials and Methods

### Strains and genetics

Worms were grown on OP50 Escherichia coli-seeded nematode growth medium plates at 20°C. Strains used in this work include: N2 (wild type reference), *ire-1(dz176)*, *ire-1(ok799)*, *ire-1(zc14)*, *xbp-1(tm2457)*, *xbp-1(zc12)*, *pek-1(ok275)*, *atf-6(ok551)*, *daf-2(e1370)*, *daf-16(mu86)*, *trf-1(nr2014)*, *kgb-1(um3) kgb-2(gk361) jnk-1(gk7)*. PVD neurons were visualized by the integrated transgene *wdIs52* (*Is[F49H12*.*4*::*GFP]*). Transgenic strains for cell-specific rescue were established by injecting the respective plasmids at 5–10 ng/μl together with *rol-6(su1006)* or *Pttx-3*::mCherry (labeling the interneuron AIY) at 50 ng/μl as an injection marker into *ire-1(ok799); wdIs52*. The PVD::DMA-1::GFP translational fusion was a kind gift of K. Shen (Stanford U, California). For a complete strain list see Supporting Information.

### Molecular cloning

The *ire-1* cDNA was amplified with gene specific primers from a N2 mixed stage cDNA sample and cloned *Kpn*I/*Sph*I downstream of the *Pttx-3*^promB^ regulatory element [58]. For the cell specific heterologous rescue the *ire-1* cDNA was placed under control of the *Pdpy-7* (hypodermis-specific), *Pmyo-3* (muscle), *Pges-1* (intestine), *Prgef-1* (pan-neuronal) or *Pser-2*^*prom3*^ promoter (PVD/OLL specific). For further details see Supporting Information.

### *xbp-1* splicing

On day 1 of adulthood, animals were collected for RNA extraction, purification and reverse transcription, using random 9-mers and standard protocol. A primers set encompassing the noncanonical intron of the *xbp-1* transcript was used, giving rise to two PCR products of amplified spliced and unspliced *xbp-1* transcript (primers: 5’- TCCGCTTGGGCTCTTGAGATGTTC-3’ and 5’-TGTCGTCGTCGGAGGAGAGGATCG- 3’). PCR products were visualized on a 2% agarose gel stained with ethidium bromide.

### Fluorescence microscopy and quantification

Images of immobilized animals (1–5 mM levamisol, Sigma) were captured using either a Zeiss Axioimager Z1 Apotome at 40X, where *Z* stacks were collected and maximum projections were used for imaging of dendrites, or with a CCD digital camera using a Nikon 90i fluorescence microscope at 20X magnification. For DMA-1::GFP signal quantification the NIS element software was used to quantify sum and mean fluorescence intensity as measured by intensity of each pixel in the selected area.

### Dissociated hippocampal cultures

Hippocampal neurons were prepared from rats at E18 as previously described [59] with modifications. In brief, dissected hippocampi were incubated in 0.05% trypsin at 37°C for 20 minutes (Invitrogen 25300054) and plated at a density of 60,000 cells per 12 mm coverslip coated with poly-l-lysine (Sigma P1274). Cells were incubated in a cell culture incubator maintained at 37°C with 5.0% CO_2_. Cytosine arabinoside (ara-c, Sigma C1768) was added at a final concentration of 2 μM at 2 days in vitro (DIV) to prevent glia cell overgrowth before being replaced with Neurobasal without ara-c at 4DIV. Neurons were transfected at 5-6DIV using Lipofectamine LTX and Plus Reagent (ThermoFisher). For cytoplasmic labeling of neurons to visualize dendrites, 60,000 cells were transfected with 0.25 μg pCAGGS-mCherry [60]. Neurons were treated at 8DIV with IRE-1 RNAse inhibitor 4μ8C (EMD Millipore 412512). 4μ8C was first dissolved in DMSO (Invitrogen) and diluted in supplemented Neurobasal. Diluted 4μ8C at 100 μM was added to cultures at 1:1 with conditioned neural media with final concentrations of 50 μM 4μ8C and 0.5% DMSO. Additional 4μ8C was added to neurons at 10DIV resulting in a final concentrations of 37.5 μM 4μ8C and 0.5% DMSO. Vehicle neurons were treated in identical ways using media containing DMSO only.

### Immunostaining, imaging, and analysis of dissociated hippocampal cultures

At 8 or 12 DIV coverslips with neurons were quickly washed two times with PBS, followed by fixation for 15 min with 4% PFA / 4% sucrose in PBS at RT. Cells were blocked and permeabilized with 3% horse serum and 0.05% Triton X-100 in PBS for 1 h at RT. Cells were incubated with antibodies against mCherry (Rockland 600-401-379) at 4°C overnight. The next day cells were washed three times with PBS and labeled with AlexaFluor-conjugated secondary antibodies (Invitrogen; 1:500) for 1 hr at RT. Cells were washed three times with PBS, stained with DAPI, and mounted on slides Aqua-Mount mounting media (Thermo Scientific). Tiled images of dendritic arbors were acquired using a Keyence BZ-X710 Fluorescence Microscope equipped with a Nikon 60X oil-immersion 1.40 NA objective. Merged composite images of the individually acquired tiled images were generated using Keyence software. Dendritic arbors were traced using the NeuronJ plugin for ImageJ [61]. Total dendritic branch length was calculated as the sum of the length of all dendrites. Average dendrite branch length is average length of each dendritic branch excluding primary dendrites, as primary dendrite lengths are highly variable across neurons. All images were acquired and all analysis was performed with the experimenter blind to conditions. Data analysis was performed using GraphPad Prism 6.

### Statistical analysis

Error bars represent the standard error of the mean (SEM) of at least 3 independent experiments. P values were calculated using the unpaired Student's t test, or one-way ANOVA with the Tukey correction for multiple comparisons (GraphPad Prism 6).

## Supporting Information

S1 Supporting Information This contains a complete strain list, as well as additional details with regard to the construction DNA constructs and transgenic strains. (DOCX)

S1 Fig IRE-1 functions cell-autonomously to pattern sensory dendrites. A.–B. Fluorescent micrographs of PVD in non-transgenic *ire-1(dz176)* mutants (A) and in *ire-1(dz176)* mutants harboring a transgene with a fosmid that contains the wild type *ire-1* locus. PVD visualized by the *wdIs52* transgene (*Is[F49H12*.*9*::*GFP]*. Arrowheads indicate cell bodies. Anterior is to the left in all panels and ventral down; scale bars indicate 20 μm.C. Quantification of defects in the genotypes indicated. Defects are defined as absence of complete menorah-like dendrites between the vulva and the anterior end of the animals.D. Quantification of PVD defects in mutant *ire-1(ok799)* animals using transgenic rescue of *ire-1* driven by tissue specific heterologous promoters (*Prgef-1*: pan neuronal expression, *Pges-1*: intestinal expression, *Pdpy-7*: hypodermal expression and *Pser-2*^*prom3*^ *short*: expression in the PVD and OLL neurons). Transgenic animals (T) and non-transgenic animals (NT) are shown side by side.E. Quantification of secondary, tertiary, and quaternary branch aggregate length. Data are represented as mean ± SEM.
(TIF)
