# Supplementary material for: Reduced Insulin/Insulin-Like Growth Factor Receptor Signaling Mitigates Defective Dendrite Morphogenesis in Mutants of the ER Stress Sensor IRE-1
Source: PLoS Genet. 2017 Jan 23;13(1):e1006579. doi: 10.1371/journal.pgen.1006579 (PMC5293268; doi:10.1371/journal.pgen.1006579)
Supplement: S1 Supporting Information — (DOCX) [file pgen.1006579.s001.docx]

**Supporting Information to**

**Reduced insulin/insulin-like growth factor receptor signaling mitigates defective dendrite morphogenesis in mutants of the ER stress sensor IRE-1**

Yehuda Salzberg^1,2^, Andrew Coleman^3^, Kevin Celestrin^1^, Moran Cohen-Berkman^2^, Thomas Biederer^3^, Sivan Henis-Korenblit^2*^ and Hannes E. Bülow^1,4*^

^1^Department of Genetics, ^4^Dominick P. Purpura Department of Neuroscience, Albert Einstein College of Medicine of Yeshiva University, Bronx, New York, 10461

^2^The Mina and Everard Goodman Faculty of Life Sciences, Bar-Ilan University, Ramat-Gan, Israel, 5290002

^3^Department of Neuroscience, Tufts University School of Medicine, Boston, MA 02111, USA

^*^ Corresponding authors:

Hannes E. Bülow

Telephone 718 430 3621

Fax 718 430 8778

e-mail: [hannes.buelow@einstein.yu.edu](mailto:hannes.buelow@einstein.yu.edu)

Sivan Henis-Korenblit

Telephone 972 3 531 8961

Fax 972 3 738 4058

e-mail: [sivan.korenblit@biu.ac.il](mailto:sivan.korenblit@biu.ac.il)

**Supporting Material And Methods**

***Strain list***

***Fluorescent reporter strains***

PVD: *wdIs52* [*PF49H12.4*::GFP] II (kind gift of David Miller)

PVD: *wyIs378* [*Pser-2prom3*::MYR-GFP *+ Prab-3*::MYR-mCherry] X (kind gift of Kang Shen)

PVD: *dzIs53* [*PF49H12.4*::mCherry] II

DMA-1 reporter*: wyEx4286* [*Pser2prom3::dma-1::gfp; Podr-1::rfp*] (kind gift of Kang Shen)

***Strains created for this study:***

EB1312*: ire-1(dz176)wdIs52II*

EB1983*: ire-1(ok799)II; wyIs378X*

EB1820*: xbp-1(zc12)III; wyIs378X*

EB1824*: atf-6(ok551)X; wdIs52II*

EB1826*: pek-1(ok275)X; wdIs52II*

SHK348 *trf-1(nr2014); wdIs52II*

SHK349 *kgb-1(um3) kgb-2(gk361) jnk-1(gk7); wdIs52II*

SHK366*: ire-1(ok799)wdIs52II*

SHK367: *ire-1(zc14)wdIs52II*

SHK368: *xbp-1(tm2457)III; wdIs52II; him-5(ok1896)V*

SHK369: *ire-1(ok799)II; daf-2(e1370)III; wyIs378X*

SHK370:*ire-1(ok799)wdIs52II; daf-2(e1370)III; daf-16(mu86)I*

SHK371: *daf-16(mu86); wdIs52II*

SHK372: *daf-2(e1370)III; wdIs52II*

SHK373: *ire-1(ok799)II; wyEx4286*

SHK374: *xbp-1(tm2457)III; wyEx4286*

SHK375: *dma-1(tm5159)I; wdIs52II*

SHK376: *ire-1(ok799)wdIs52II; dma-1(tm5159)I*

SHK377: *ire-1(ok799)II; daf-2(e1370)III; dma-1(tm5159)I*

SHK378: *ire-1(ok799)II; daf-2(e1370)III; wyEx4286*

SHK379: *daf-2(e1370)III; wyEx4286*

***Transgenic strains***

**Heterologous rescue of *ire-1* PVD dendrite branching defect**

The *ire-1* cDNA was cloned under control of heterologous promoters: hypodermal *Pdpy-7* , intestinal *Pges-1*, pan-neuronal *Prgef-1*, PVD/OLL-specific *Pser-2prom3* and an AIY-specific *Pttx-3* promoter. All constructs were injected at 5-10 ng/µl into *ire-1(dz176) wdIs52II*, together with the *Pttx-3::mCherry* marker at 50 ng/µl and *pBluescript* at 40 ng/µl*.*

**Fosmid rescue of *ire-1* PVD dendrite branching defect**

Fosmid WRM0631bG07 was injected into *ire-1(dz176)wdIs52II* animals at 2ng/µl with 50ng/µl *P*myo-3*::mCherry* as marker and *pBluescript* at 40 ng/µl*.*

**List of plasmids used in transgenic experiments**

| **Plasmids for transgenesis** | **Source** |
| --- | --- |
| *Pttx-3::ire-1* | *ire-1* ORF was amplified from N2 cDNA and cloned KpnI/SphI into *Pttx-3* backbone |
| *Prgef-1::ire-1* | *ire-1* cDNA was cut from *Pttx-3::ire-1 and* cloned into *Prgef-1* into XmaI/ApaI sites |
| *Pdpy-7::ire-1* | *ire-1* cDNA was cut from *Pttx-3::ire-1 and* cloned into *Pdpy-7* into XmaI/AflII sites |
| *Pser2prom3::ire-1* | *ire-1* cDNA was cut from *Pttx-3::ire-1 and* cloned into *Pser2prom3* into XmaI/ApaI sites |
| *Prgef-1::ire-1^Δlum^* | Site directed mutagenesis on *Prgef-1::ire-1* using QuickChange kit (Agilent) |
| *Prgef-1::ire-1^K853A^* | Site directed mutagenesis on *Prgef-1::ire-1* using QuickChange kit (Agilent) |
| *Prgef-1::ire-1 ^K853AΔlum^* | Site directed mutagenesis on *Prgef-1::ire-1* using QuickChange kit (Agilent) |
| *Prgef-1::ire-1^L589G^* | Site directed mutagenesis on *Prgef-1::ire-1* using QuickChange kit (Agilent) |
| *Prgef-1::ire-1^R882D^* | Site directed mutagenesis on *Prgef-1::ire-1* using QuickChange kit (Agilent) |

**List of transgenic strains with genotypes and strain names**

| **Strain name** | **Constructs** | **Genotype** | **Line^a^** |
| --- | --- | --- | --- |
| SHK380 | *Prgef-1::ire-1* and *Pttx-3::mCherry* | *biuEx16; ire-1(ok799) wdIs52II* | *1* |
| SHK381 | *Prgef-1::ire-1* and *Pttx-3::mCherry* | *biuEx17; ire-1(ok799) wdIs52II* | *2* |
| SHK382 | *Prgef-1::ire-1 ^K853A^* and *Pttx-3::mCherry* | *biuEx18; ire-1(ok799) wdIs52II* | *1* |
| SHK383 | *Prgef-1::ire-1 ^K853A^* and *Pttx-3::mCherry* | *biuEx19; ire-1(ok799) wdIs52II* | *2* |
| SHK384 | *Prgef-1::ire-1^Δlum^* and *Pttx-3::mCherry* | *biuEx20; ire-1(ok799) wdIs52II* | *1* |
| SHK385 | *Prgef-1::ire-1^Δlum^* and *Pttx-3::mCherry* | *biuEx21; ire-1(ok799) wdIs52II* | *2* |
| SHK386 | *Prgef-1::ire-1^Δlum K853A^* and *Pttx-3::mCherry* | *biuEx22; ire-1(ok799) wdIs52II* | *1* |
| SHK387 | *Prgef-1::ire-1^Δlum K853A^* and *Pttx-3::mCherry* | *biuEx23; ire-1(ok799) wdIs52II* | *2* |
| SHK388 | *Prgef-1::ire-1^L589G^* and *Pttx-3::mCherry* | *biuEx24; ire-1(ok799) wdIs52II* | *1* |
| SHK389 | *Prgef-1::ire-1^L589G^* and *Pttx-3::mCherry* | *biuEx25; ire-1(ok799) wdIs52II* | *2* |
| SHK390 | *Prgef-1::ire-1^R882D^* and *Pttx-3::mCherry* | *biuEx26; ire-1(ok799) wdIs52II* | *1* |
| SHK391 | *Pser2prom3::ire-1* and *Pttx-3::mCherry* | *biuEx27; ire-1(ok799) wdIs52II* | *1* |
| SHK392 | *Pser2prom3::ire-1* and *Pttx-3::mCherry* | *biuEx28; ire-1(ok799) wdIs52II* | *2* |
| SHK393 | *Pser2prom3::ire-1* and *Pttx-3::mCherry* | *biuEx29; ire-1(ok799) wdIs52II* | *3* |
| SHK394 | *Pges-1::ire-1* and *Pttx-3::mCherry* | *biuEx30; ire-1(ok799) wdIs52II* | *1* |
| SHK395 | *Pdpy-7::ire-1* and *Pttx-3::mCherry* | *biuEx31; ire-1(ok799) wdIs52II* | *1* |
| SHK396 | *Pdpy-7::ire-1* and *Pttx-3::mCherry* | *biuEx32; ire-1(ok799) wdIs52II* | *2* |
| EB1821a | WRM0631bG07 and *Pmyo-3::mCherry* | *biuEx33; ire-1(dz176) wdIs52II* | *1* |
| EB1821b | WRM0631bG07 and *Pmyo-3::mCherry* | *biuEx34; ire-1(dz176) wdIs52II* | *2* |

^a^ line refers to the respective numbering of extrachromosomal transgenic lines used.
