# Supplementary figures and images for: Reduced Insulin/Insulin-Like Growth Factor Receptor Signaling Mitigates Defective Dendrite Morphogenesis in Mutants of the ER Stress Sensor IRE-1
Source: PLoS Genet. 2017 Jan 23;13(1):e1006579. doi: 10.1371/journal.pgen.1006579 (PMC5293268; doi:10.1371/journal.pgen.1006579)

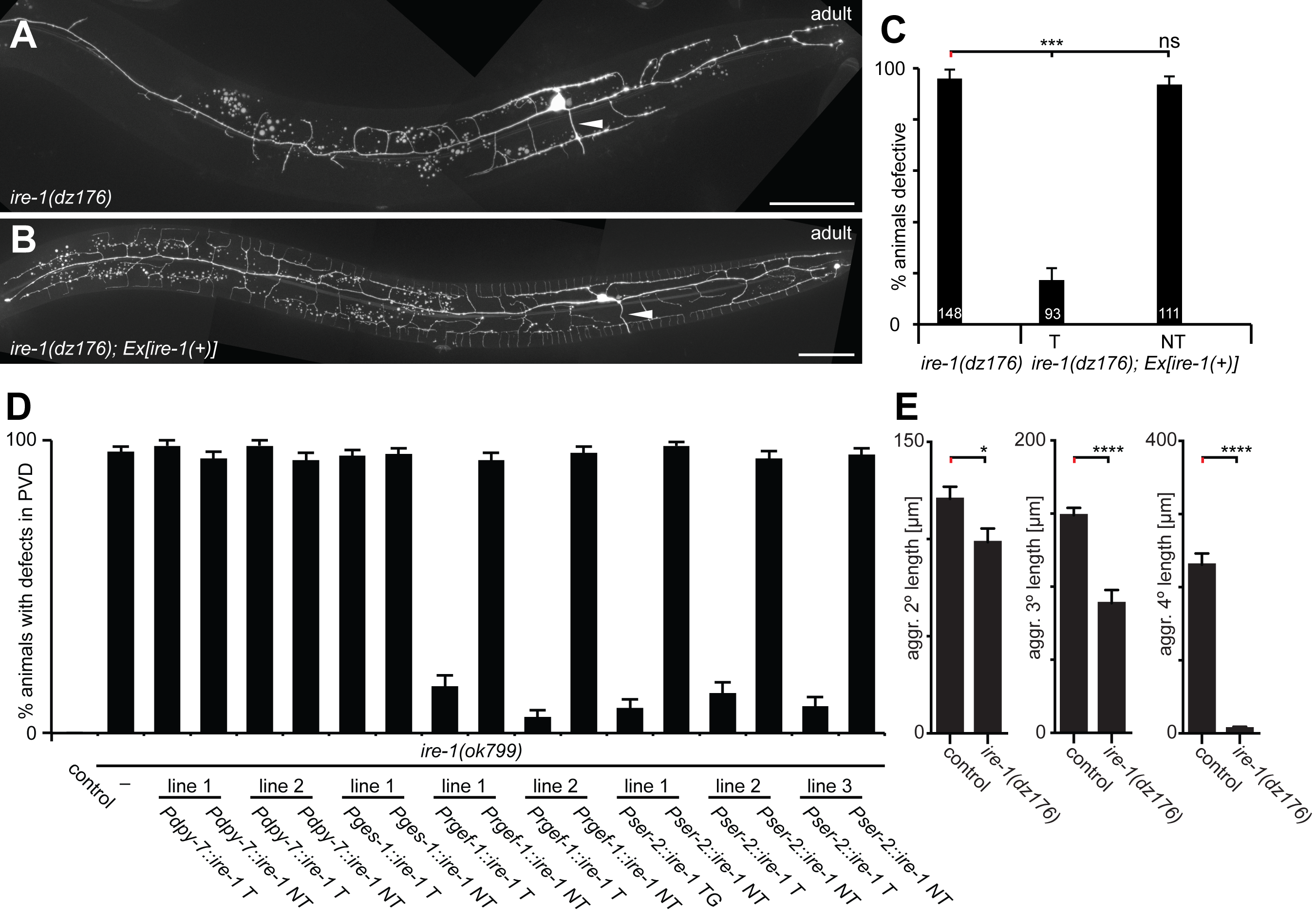

Supplement: S1 Fig — A.–B. Fluorescent micrographs of PVD in non-transgenic ire-1(dz176) mutants (A) and in ire-1(dz176) mutants harboring a transgene with a fosmid that contains the wild type ire-1 locus. PVD visualized by the wdIs52 transgene (Is[F49H12.9::GFP]. Arrowheads indicate cell bodies. Anterior is to the left in all panels and ventral down; scale bars indicate 20 μm.C. Quantification of defects in the genotypes indicated. Defects are defined as absence of complete menorah-like dendrites between the vulva and the anterior end of the animals.D. Quantification of PVD defects in mutant ire-1(ok799) animals using transgenic rescue of ire-1 driven by tissue specific heterologous promoters (Prgef-1: pan neuronal expression, Pges-1: intestinal expression, Pdpy-7: hypodermal expression and Pser-2prom3 short: expression in the PVD and OLL neurons). Transgenic animals (T) and non-transgenic animals (NT) are shown side by side.E. Quantification of secondary, tertiary, and quaternary branch aggregate length. Data are represented as mean ± SEM. (TIF) [file pgen.1006579.s002.tif]
